# Field Evaluation of Outdoor Ultra-Low Volume (ULV) Applications against Phlebotomine Sand Flies (Diptera: Psychodidae) in Al Rabta, North-West of Libya

**Published:** 2017-09-08

**Authors:** Mostafa Ramahdan Dokhan, Mohamed Amin Kenawy, Taher Shaibi, Badereddin Bashir Annajar

**Affiliations:** 1 National Center for Disease Control, Ministry of Health, Tripoli, Libya; 2 Zoology Department, Faculty of Sciences, Sabratha, University of Zawia, Zawia, Libya; 3 Department of Entomology, Faculty of Science, Ain Shams University, Abbassia, Cairo, 11566, Egypt; 4 Zoology Department, Faculty of Science, University of Tripoli, Tripoli, Libya; 5 Public Health Department, Faculty of Medical Technology, University of Tripoli, Tripoli, Libya

**Keywords:** Sand flies, ULV application, CL cases, Libya

## Abstract

**Background:**

Al Rabta is a rural area in the North-West of Libya that represents an important focus of zoonotic cutaneous leishmaniasis. This study aimed to evaluate the effect of Ultra Low Volume (ULV) applications in controlling sand flies and its impact on leishmaniasis transmission in this area.

**Methods:**

Two neighboring villages were selected: Al Rabta West (RW) as cypermethrin treated village and Al Rabta East (RE) as check one. The ULV was evaluated through 3 spraying cycles during Apr, Jun and Sep 2013. In the two villages, a number of outdoor sites were selected for sampling of sand flies (twice a month) using the CDC light traps. The cases of CL reported in the two villages during the study period were obtained from Al Rabta health center.

**Results:**

The two villages were similar where 9 species of sand flies (6 of *Phlebotomu* and 3 of *Sergentomyia*) were collected of which *S. minuta* and *P. papatasi* were the abundant species. As compared to the pre- ULV spraying, during the post- spraying periods: i) the reduction in abundance of the different species ranged from 20.85 to 77.52% with 46.69% as an overall reduction for all species altogether and, ii) in significantly (P> 0.05) higher mean ratio of males: females for all species altogether (1:2.41). Moreover, ULV spraying resulted in the absence of CL (*Leishmania major*) cases (Passive Case Detection)

**Conclusion:**

The efficiency of ULV spraying in reducing sand fly population, CL cases and consequently limits the disease transmission.

## Introduction

The current control measures against leishmaniasis rely on chemotherapy to alleviate disease and on vector control to reduce transmission (1). To date, there is no vaccine for routine use against leishmaniasis.

The main control methods for sand fly vectors were reviewed including insecticide spraying (houses, the peridomestic environment, and resting sites), environmental management, personal protection, integrated control and biological control (2–5). These methods were applied and evaluated by several workers (6–12). In general, sand fly control is complicated by the many species of sand flies and reservoir hosts involved in the transmission of multiple *Leishmania* species over a variety of geographic habitats (3).

Regarding the Ultra-Low Volume (ULV) applications, such application was evaluated against sand fly species in Equatorial Kenya and reported that sampling of the wild populations before and after treatments suggested local population suppression of sand fly from ULV treatments, as well as a possible repellent effect in nearby untreated areas (13). However, various insecticide applications, including ULV, did not have a significant effect against sand flies in southern Iraq (14). Overall, 21 ULV spray operations were conducted in the Al Anbar provincial capital of Ramadi, Iraq during five months of the sand fly season and stated that “Based on our surveillance program, we do not know whether our ULV missions had any impact on sand fly populations” (15).

In Libya, cutaneous leishmaniasis (CL) is an endemic disease since 1910 when it was detected for the first time (16). Several cases were reported and exclusively originated from the North-Western (NW) districts of the country (17, 18). Zoonotic Cutaneous Leishmaniasis (ZCL) due to *L. major* was confirmed (19–21) and is largely the main form in this country (22). Only two reports (19, 23) concerning the implication of *Phlebotomus papatasi* and *P. sergenti* as suspected vectors of CL in NW region. Moreover, in only three occasions, *P. papatasi* and *P. longicuspis* were found positive for *Leishmania* spp. (16, 20, 24).

The national program to combat leishmaniasis in Libya was established in Nov 2006. The program was launched to achieve the main objectives of limiting the spread of the disease to new areas and treatment of infected cases in endemic areas. The initial phase of the program management started in 2007 to intensify efforts and give priority to reduce the epidemic of explosions that have occurred in the Tawergha area through chemical control of wild rodents (the main reservoirs of ZCL, *L. major*). The second phase (2008) included an expanded campaign against sand flies in all infested areas through residual-spraying of outdoor resting sites (Unpublished reports of the national control program of leishmaniasis, NCPL, National Center for Disease Control, NCDC, Ministry of Health, MOH, Tripoli, Libya).

Al Rabta in the NW of Libya is one of foci where CL is endemic for a long time with huge outbreak occurred during 1977– 1980 and 2004–2012 (Unpublished report of the NCPL, NCDC, MOH, Tripoli, Libya 2013). For this, the present study was carried out to evaluate the effect of ULV applications in controlling sand flies and its impact on CL transmission in this area.

## Materials and Methods

### Study area

**Fig. 1:**
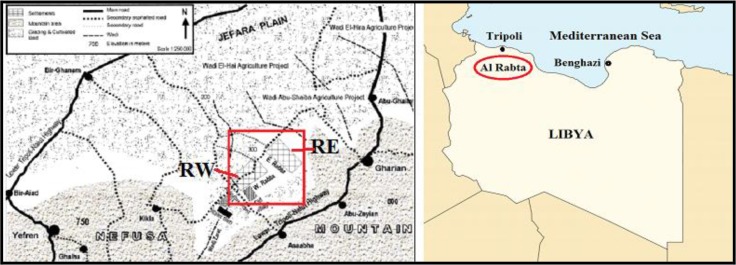
Location of Al Rabta area in the northern-west of Libya

The study was carried out from Apr to Sep 2013 in two neighboring villages (about 3km apart from each other) in Al Rabta area: Al Rabta East (RE) and Al Rabta West (RW) (Fig. 1). Al Rabta is a rural area located 80 km south of Tripoli in the foothill of Nafusa Mountain (32°9′46.59″N, 12°50′50.65″E) with an altitude of about 300m above sea level and a population of about 6000 inhabitants (2010 estimates). Most people in the area practice farming and animal rearing. Around the houses are shelters for domestic animals made of brick and fruit trees. The area is characterized by a warm and dry climate with an average annual rainfall of 16mm. The mean annual temperature of the area is 21 °C. January is usually the coldest month, while Aug is the warmest month. The summer temperatures can exceed 45 °C. The rainy season is from Nov to Feb and Jul being the driest month (http://www.libya.climatemps.com).

In the two villages, a number of outdoor sites (6 in RE and 7 in RW) at a distance of 300m from each other were selected for collecting of sand flies. These sites were selected where rodent burrows were numerous.

### Ultra-Low Volume application

The RW village was selected as the insecticide treated area while the RE was selected as the check village that received no insecticide application. The treated area was sprayed with cypermethrin™ 10.0% EC, at a dilution of 1L per 200L of water and at a rate of about 350L per hectare. The area received 3 spraying cycles (Table 1) in Apr (17–25), Jun (16–21) and Sep (15–19). The whole area around the sand fly sampling sites (100 hectares/cycle) was sprayed including ground, trees, old settlements, caves, etc.

**Table 1.** Dates of ULV spraying cycles and sand fly collections before and after the spraying cycles (2013)

**Period**	**Apr**	**Jun**	**Sep**
Pre-spraying collection	14–16	13–15	12–14
ULV spraying	17–25	16–21	15–19
Post-spraying collection	26–28	22–24	20–22

### Sand fly collection and processing

Sand flies were collected for three consecutive nights (twice a month) during the study period from Apr to Sep 2013 using the CDC (Center for Disease Control) light traps (Model 512, John W. Hock, and Gainesville, FL, USA). Except for the 3 ULV cycles, the sand fly collection was carried out directly before and after the spraying cycle (Table 1). The traps (6 and 7 traps/ night for RE and RW, respectively) were set before sunset and collected next morning. Traps were hanged on approximately 30cm from the ground. The collected sand flies were aspirated, placed in labeled paper cups that kept in a picnic icebox while being transported to the field laboratory. In the laboratory, flies were preserved in 70% alcohol and then mounted in Puri’s medium. Flies were sexed and identified using morphological keys (16, 25).

### Leishmaniasis cases

The cases of CL reported in the two villages during the study period (Passive Case Detection, PCD) were obtained from the respective Al Rabta health center.

### Statistical analysis

The means SD’s for the obtained data were calculated and analyzed by one-way Analysis of Variance (ANOVA). The Chi-squared (χ^2^) analysis was used to test the deviation of the resulting fly sex ratios (male: female) from the expected 1:1 ratio. The SSP (26) was used for statistical analysis.

## Results

A total of 1594 and 2332 sand flies of 9 species (6 of *Phlebotomu* and 3 of *Sergentomyia*) were collected in RW and RE, respectively during the study period (Apr–Sep 2013) of which *S. minuta* (32.50% and 26.20% in RW and RE, respectively). *Phlebotomus papatasi* (22.21% and 23.03% in RW and RE, respectively) were the most abundant species (Table 2). These were followed in descending order of abundance by *S. fallax*, *P. alexandri*, *P. longicuspis*, *P. sergenti*, *P. chabaudi*, *P. langeroni* and *S. antennata* in the two villages.

**Table 2.** Relative abundance of sand flies collected in Al Rabta West (RW) and Al Rabta East (RE) from April to September 2013

**Species**	**RW**		**RE**	

	**NO**	**%**	**NO**	**%**
***Phlebotomus***				
*P. papatasi*	354	22.21	537	23.03
*P. alexandri*	172	10.79	242	10.38
*P. longicuspis*	130	08.16	220	09.43
*P. sergenti*	116	07.28	177	07.59
*P. chabaudi*	046	02.89	099	04.25
*P. langeroni*	025	01.57	053	02.27
***Sergentomyia***				
*S. minuta*	518	32.50	611	26.20
*S. fallax*	222	13.93	358	15.35
*S. antennata*	11	00.69	035	01.50

**Total**	**1594**		**2332**	

The effect of the outdoor ULV spraying on the densities of the different sand fly species (mean number of flies per collection sites, n= 7 sites) was examined in RW through 3 spraying cycles during Apr, Jun and Sep 2013. The comparable figures in RE as check village (n= 6 sites) were also examined during the same period (Table 3). The compiled mean of the three cycles was calculated and revealed that in RW, significantly lower densities were found during post spraying than those during pre-spraying (38.91–52.78% reduction) for *P. papatasi* P< 0.001, *P. longicuspis* P< 0.01, *P. sergenti* P< 0.05, *S. minuta* P< 0.001, *S. fallax* P< 0.001 and for all sand fly species altogether (46.69% reduction, P< 0.001). Besides, lower densities were found during post spraying for *P. alexandri*, *P. chabaudi*, *P. langeroni*, and *S. antennata* than those during the pre-spraying (20.85–77.52% reduction) however, the difference was not significant (P> 0.05). In RE, the comparable fly numbers/site for pre- and post-spraying were insignificantly different (P> 0.05) for all sand fly species. Comparison of the total fly densities in the two villages indicated that significantly (F= 7.72, df= 1, 11, P< 0.05) lower pre-spraying density in RW (75.57 fly/site) than in RE (107.00 flies/site). Similarly, significantly (F= 46.54, df= 1, 11, P< 0.05) lower post-spraying density was observed in RW (40.29 fly/site) than in RE (104.17 fly/site).

**Table 3.** Mean density of sand flies (fly/collection site) pre- and post-ULV spraying after three spraying cycles in Al Rabta West (RW) from April to September 2013

**Species**	**Treated: Al Rabta West (RW)**				**Check: Al Rabta East (RE)**		

	**Pre-^a^**	**Post-^a^**	**F_(1,12)_^b^**	**% R^c^**	**Pre-^a^**	**Post-^a^**	**F_(1,10)_^b^**
*P. papatasi*	15.43	09.29	20.54***	39.78	026.67	023.17	0.86^NS^
*P. alexandri*	06.14	04.86	01.94^NS^	20.85	009.33	008.83	0.09^NS^
*P. longicuspis*	06.57	03.29	11.58**	49.92	009.67	009.50	0.01^NS^
*P. sergenti*	05.14	03.14	05.65*	38.91	007.50	008.00	0.08^NS^
*P. chabaudi*	02.86	01.29	04.65^NS^	54.90	005.00	005.67	0.19^NS^
*P. langeroni*	01.29	00.29	03.87^NS^	77.52	002.50	002.50	0.00^NS^
*S. minuta*	25.71	12.14	53.61***	52.78	028.17	025.00	0.93^NS^
*S. fallax*	10.86	05.14	31.37***	52.67	016.33	016.67	0.01^NS^
*S. antennata*	01.29	00.86	01.23^NS^	33.33	001.83	001.33	0.74^NS^

**All species**	**75.57**	**40.29**	64.95***	**46.69**	**107.00**	**104.17**	**0.03^NS^**

a SD’s were omitted,

b NS: not significant (P> 0.05),

* P< 0.05,

** P< 0.01,

*** P< 0.001,

c R: Reduction

The effect of the 3 ULV spraying cycles on the overall sex ratios of sand fly species in RW was examined (Table 4) and revealed that for pre-spraying periods, all ratios do not deviate from the expected 1:1 ratio (P> 0.05) except for *P. alexandri* (P< 0.05), *P. longicuspis* (P< 0.01), *P. sergenti* (P< 0.05) and *P. chabaudi* (P< 0.05). For post-spraying periods, all ratios were significantly deviated from the expected 1:1 ratio in favor of males except that of *S. fallax* (P> 0.05). The overall mean ratio for all species altogether indicated higher post-spraying ratio (1:2.41) than that of pre-spraying (1:1.52), however, means were not significantly different (F= 1.61, df= 1, 14, P< 0.05).

**Table 4.** Numbers and sex ratios (male: 1 female) of sand flies pre- and post-ULV spraying in Al Rabta West (RW) from April to September 2013

**Species**	**Pre-**			**Post-**		

	**M**	**F**	**M:1F**	**M**	**F**	**M:1F**
*P. papatasi*	060	059	1.02^NS^	043	022	1.95*
*P. alexandri*	029	021	2.42*	021	013	1.62*
*P. longicuspis*	035	011	3.18**	017	006	2.83*
*P. sergenti*	024	012	2.00*	019	003	6.33**
*P. chabaudi*	013	007	1.86*	006	003	2.00*
*P. langeroni*	003	006	0.50^NS^	002	000	----
*S. minuta*	102	078	1.31^NS^	053	032	1.66*
*S. fallax*	036	042	0.86^NS^	012	024	0.50 ^NS^
*S. antennata*	003	006	0.50^NS^	001	000	----

**Total**	**305**	**242**	**1.26^NS^**	**174**	**103**	1.69*

**Mean±SD**	**1.52 ±0.92**			**2.41 ±1.86**		

**NS:** not significant (P> 0.05),

* P< 0.05,

** P< 0.01 (χ^2^-test)

The results of reported CL cases (PCD) indicated that during the study period, no cases were reported in RW in comparable to three cases in RE (one each in Apr, Aug and Sep 2013).

## Discussion

Nine sand fly species (*P. papatasi*, *P. alexandri*, *P. longicuspis*, *P. sergenti*, *P. chabaudi*, *P. langeroni*, *S. minuta*, S*. fallax* and *S. antennata*) were collected in RE and RW in this study. *Phlebotomus papatasi* is the most important as the main vector of *L. major*, the causative agent of ZCL previously isolated from such fly species in NW (16, 22). The same species in addition to *S. clydei*, *S. christophrsi* and one unidentified *P.* (*Larroussius*) sp were previously reported in Al Rabta (16). Overall, 1594 and 2332 sand flies were collected during the study period in RW and RE, respectively of which *S. minuta* and *P. papatasi* were the most abundant species in both villages. Both *P. langeroni* and *S. antennata* were rare. Almost similar results mainly for *P. papatasi* were obtained (16, 17, 27) and investigated sand flies in some NW areas.

Leishmaniasis represents a major public health problem in several countries of the Eastern Mediterranean Region (EMR) of the WHO (28) including Libya. Two forms of cutaneous leishmaniasis exist in Libya, these are ZCL (*L. major*) and anthroponotic cutaneous leishmaniasiss (ACL, *L. tropica*)*.* Aoun and Bouratbine (22) reviewed the situation of leishmaniasis in Libya and stated “Most published reports in Libya concern ZCL, which is largely the main form in this country”. The main ZCL foci are located in the NW of the country (17). Due to lack of scientifically based control program, and due to the wide spread of animal reservoirs (the rodent *Psammomys libycus* and *Meriones obesus*) the disease was largely extended to other new areas. According to the report of the national NCDC, MOH, Tripoli, Libya (2008), about 30000 ZCL cases were reported during the last 30 years. During 2005–2008, about 6000 cases of which 1800 in 2008 were reported (28).

Several methods exist at present for leishmaniasis control used individually or in combination. The selection of the method or combination of methods depends on the type of the leishmaniasis to be controlled and the method should be situation specific (4). The main sand fly vector control methods were reviewed and evaluated (3–12).

The control efforts of the sand fly vectors of leishmaniasis are problematic and directed only to adults. This is because their larvae develop in largely unknown terrestrial habitats making them impervious to available control measures (3, 5). However, the diversity of phlebotomine biology and ecology makes it very difficult to adapt one control strategy for all endemic areas. It can be effective in reducing the transmission of the disease but mainly in places where the vector is endophilic and peridomestic (16). In certain areas, effective control has been achieved as a side effect of malaria control programmes (29–31).

Depending on application techniques, timing and target species, sand flies are known to be highly susceptible to insecticides (3, 32–37) and only a few cases of *P. papatasi* resistance to DDT have been reported (38, 39). However, the prolonged contact with insecticides might lead to the appearance of resistance (40) that needs to assess the potential of sand flies to develop resistance that could cause problems in control campaigns (2).

The space spraying with ULV is widely used to control sand flies, but few rigorous studies have evaluated its efficacy (41). In this study, the application of 3 ULV spraying cycles with cypermethrin resulted in reduction of sand fly densities that ranged from ca 21% to 78% for the 9 species and an overall reduction for all species altogether of ca 47% (P< 0.001). Such results indicate the efficiency of ULV spraying in reducing sand fly population. Sampling of wild populations (*P. duboscqi*) in western Kenya before and after treatments suggested local population suppression from ULV treatments (13). “Sand flies in Libya are most active on warm, clear nights with little wind as the case in Iraq” (42). Such conditions are also favorable for applying ULV-based adulticides (41), however, various insecticide applications, including ULV, did not have a significant effect against sand flies in southern Iraq (14). Moreover, 21 ULV spray operations were conducted in Camp Ramadi, Iraq during five months of the sand fly season (Apr to Aug 2009) (15) and although they obtained lower mean catch/night after spraying (0.563–2.002) than before ULV operations (0.839–2.002), however, means were not significantly different except on 25 May (P< 0.05). ”Based on our surveillance program, we do not know whether our ULV missions had any impact on sand fly populations. Our insecticide applications were part of an actual vector control program lacking an untreated area to serve as a control, which complicates the interpretation of our results”. Such contradictory results may be due to the local conditions in the study areas (weather conditions, sand fly fauna etc.).

The effect of the 3 ULV spraying cycles on the overall sex ratios (male: female) of sand flies in RW indicated that most of ratios for pre- spraying periods do not deviate from the expected 1:1 ratio (P> 0.05)*.* With the exception of *S. fallax* (P> 0.05), all post- spraying ratios were significantly deviated from the expected 1:1 ratio in favor of males. Mean ratio for all species altogether indicated higher ratio post-spraying than that pre-spraying (P< 0.05) which may indicate that ULV application affects females more than males, however, further investigations are required.

No CL cases (PCD) were reported in RW during the period of the three cycles of insecticidal treatment compared to 3 cases reported in RE. In a concurrent study (43), the reported CL cases during the sand flies activity period (Apr to Nov 2012 and 2013) in RW were 11 and 4 in the two years, respectively (ie 63.64% reduction in cases during ULV application) compared to 8 and 9 cases in RE in 2012 and 2013, respectively. However, such disappearance of Cl cases due to ULV application in this study may not be conclusive, further studies are required including active-case detection (ACD). There are no available reports on the effect of ULV on leishmaniasis incidence. For other insecticide applications however, such data exists. The impact of indoor residual spraying and impregnated bed nets were reviewed in reducing the leishmaniasis cases in several Asian countries (9). The effect of 65% permethrin spot- was examined on the prevalence of canine visceral leishmaniasis (VL) and the abundance of sand flies in two neighborhoods in Corumbá, Mato Grosso does Sul, Brazil known to have a high prevalence of VL (44). A reduction in leishmaniasis prevalence was observed. The results suggest that regular use of 65% permethrin during months of high risk for canine VL can be a useful strategy for reducing the prevalence of this disease in hyperendemic areas. In Bangladesh, the effect of a community- based intervention was evaluated with insecticide impregnation of existing bed-nets in reducing VL incidence and found that this intervention reduced VL by 66.5% (45).

## Conclusion

The stand-alone ULV spraying with cypermethrin proved effective in reducing the outdoor sand fly population by ca 47%. However, if an endophilic sand fly species involved, this needs to be supplemented by indoor insecticide applications to increase the efficiency of sand fly/*Leishmania* control operations. The situation in Libya necessitates continuation and strengthens the ongoing leishmaniasis control program.
